# A note from the Editor-in-Chief

**DOI:** 10.1515/iss-2024-0011

**Published:** 2024-03-18

Dear colleagues, dear readers,

For almost 10 years I have had the privilege to work as Editor-in-Chief for “Innovative Surgical Sciences”, the gold open access journal to the German Society of Surgery. In these 10 years we – the Editorial Board, the researchers, the reviewers and the German Society of Surgery – established the journal within the world of academic surgical publications and we are proud and happy that we gained the first impact factor of 1.3 in June, 2023.

After 10 years I have the feeling that it is time for a change. The basis for further innovations and future development has been established and now is the right time for new steps. Therefore, I decided to step down as Editor-in-Chief.

The German Society of Surgery is glad to announce that Prof. Dr. Udo Rolle, head of pediatric surgery at the university hospital Frankfurt, and PD Dr. Juliane Liese, senior staff digestive surgeon at university hospital Giessen, will take over the function as Editors-in-Chief. Both are respected and well-known experts in their surgical disciplines and we feel that a cooperative editorship is, among other aspects, the right step into the future.

Once again, I would like to thank the German Society of Surgery and the publisher De Gruyter, in particular Dr. Simone Sporn, for the trust and support in the last 10 years. It was a privilege to serve as Editor-in-Chief! All the best for the new Editors-in-Chief and of course for Innovative Surgical Sciences!

Joachim Jaehne, Hannover.

